# Effect of a heat shock protein 90-specific inhibitor on the proliferation and apoptosis induced by VEGF-C in cervical cancer cells

**DOI:** 10.3892/etm.2014.1930

**Published:** 2014-08-22

**Authors:** XUE DU, YONGMEI LI, XU JING, LINA ZHAO

**Affiliations:** 1 Department of Obstetrics and Gynecology, General Hospital, Tianjin Medical University, Tianjin 300052, P.R. China; 2 Department of Microbiology and Immunology, Tianjin Medical University, Tianjin 300052, P.R. China; 3 Department of Biological Engineering, College of Life Sciences, Hebei United University, Tangshan, Hebei 063000, P.R. China

**Keywords:** cervical cancer, heat shock protein 90, vascular endothelial growth factor-C, proliferation, apoptosis

## Abstract

The aim of the present study was to investigate the effect of heat shock protein 90 (Hsp90)-specific inhibitor geldanamycin (GA) on the proliferation and apoptosis induced by vascular endothelial growth factor-C (VEGF-C) in cervical cancer cells. HeLa cells (1×10^6^/ml) in the logarithmic growth phase were incubated without serum for 24 h. The cells were pretreated with kinase insert domain receptor antibody (KDR)-Ab (20 μg/ml), phosphatidylinositol 3-kinase (PI3K) inhibitor LY294002 (3 μmol/l), mitogen-activated protein kinase (MAPK) inhibitor PD98059 (30 μmol/l) or Hsp90-specific inhibitor GA (10 μmol/l) for 30 min, and then treated with VEGF-C (50 ng/μl) for a further 24 h. The cells were harvested for MTT analysis, annexin V-FITC/propidium iodide double staining for early apoptosis and SDS-PAGE and western blot analysis in order to determine Hsp90, B-cell lymphoma 2 (Bcl-2), Bcl-2-associated X protein (Bax) and cyclin D1 expression. Treatment with VEGF-C alone induced Hsp90 protein expression in HeLa cells at all time-points. Hsp90 expression was increased 3.31-fold in VEGF-C treated HeLa cells, and this increase was attenuated in the treatment groups (2.17-, 1.69-, 1.82-fold in VEGF-C + KDR-Ab, VEGF-C + PD98059 and VEGF-C + LY294002, respectively). The proliferation of the VEGF-C-treated HeLa cells was increased ~2.13-fold, while that of the VEGF-C + GA-treated HeLa cells decreased 0.87-fold (P<0.05). Even low concentrations of GA (0.02 μmol/l) were found to inhibit the Bcl-2 and cyclin D1 protein expression induced by VEGF-C. Therefore, the results indicate that the Hsp90-specific inhibitor GA has a critical role in the proliferation and apoptosis induced by VEGF-C in cervical cancer cells.

## Introduction

Heat shock protein 90 (Hsp90) has been identified to be a potential therapeutic target for cancer. It is an abundant molecular chaperone involved in the folding, assembly, maturation and stabilization of specific target proteins that are critical for the proliferation and survival of cancer cells. Target proteins dependent on Hsp90 have been implicated in all six hallmarks of cancer, including growth signal self sufficiency, anti-growth signal insensitivity, evasion of apoptosis, unlimited replicative potential, metastasis and tissue invasion and sustained angiogenesis (1–3). Therefore, inhibition of Hsp90 provides a combinatorial attack on multiple signaling pathways responsible for malignant cell growth. Hsp90 also has an important role in the proliferation and apoptosis of cervical carcinoma cells (4–7). The phosphatidylinositol 3-kinase (PI3K) and mitogen-activated protein kinase (MAPK) pathways are known to be involved in the vascular endothelial growth factor-C (VEGF-C)-induced proliferation and apoptosis of HeLa cells, with the overexpression of downstream genes, including B-cell lymphoma 2 (Bcl-2) and cyclin D1 (8). However, the role of Hsp90 in the proliferation and apoptosis of HeLa cells induced by VEGF-C has yet to be elucidated. Therefore, in the present study, Hsp90 expression in HeLa cells treated with Hsp90-specific inhibitor geldanamycin (GA) acting in concert with VEGF-C was investigated.

## Materials and methods

### Materials

The human cervical carcinoma cell line, HeLa, was provided by the Tianjin Institute of Hematology (Chinese Academy of Medical Science; Tianjin, China). GA was purchased from Alexis Biochemicals (San Diego, CA, USA) and Hsp90 rabbit anti-human polyclonal antibody was obtained from Cell Signaling Technology, Inc. (Beverly, MA, USA). Bcl-2, Bcl-2-associated X protein (Bax), GAPDH mouse monoclonal antibodies and cyclin D1 polyclonal antibody were obtained from Santa Cruz Biotechnology, Inc. (Santa Cruz, CA, USA). Goat anti-rabbit and goat anti-mouse secondary antibodies were purchased from ZSGB Bio. Co. (Beijing, China), and recombinant VEGF-C protein was obtained from R&D Systems (Minneapolis, MN, USA).

### Overview of methods

Hsp90 expression levels were first evaluated in VEGF-C-treated HeLa cells. HeLa cells (1×10^6^/ml) in the logarithmic growth phase were incubated without serum for 24 h, and then treated with VEGF-C (at a final concentration of 50 ng/μl) for 3, 6, 12 and 24 h. The cells were harvested and subjected to SDS-PAGE and western blot analysis in order to determine Hsp90 protein expression.

The pathways involved were then investigated. HeLa cells (1×10^6^/ml) in the logarithmic growth phase were incubated without serum for 24 h, and then pretreated with kinase insert domain receptor antibody (KDR)-Ab (20 μg/ml; Tianjin Institute of Hematology, Chinese Academy of Medical Science), PI3K inhibitor LY294002 (3 μmol/l; Promega Corp. Madison, WI, USA), MAPK inhibitor PD98059 (30 μmol/l; Promega Corp.) or Hsp90-specific inhibitor GA (10 μmol/l) for 30 min. The cells were then treated with VEGF-C (50 ng/μl) for a further 24 h. The cells were harvested and subjected to SDS-PAGE and western blot analysis for Hsp90 protein expression.

Finally, the role of Hsp90 in the proliferation and apoptosis of HeLa cells induced by VEGF-C was investigated. HeLa cells (1×10^6^/ml) in logarithmic growth phase were incubated without serum for 24 h, pretreated with GA (0.02 μmol/l) for 30 min, and then treated with VEGF-C (50 ng/μl) for 24 h. The cells were harvested for MTT analysis, annexin V-FITC/propidium iodide (PI) double staining for early apoptosis, and SDS-PAGE and western blot analysis to determine the levels of Bcl-2, Bax and cyclin D1 expression.

### MTT cell proliferation assay

HeLa cells were seeded onto 96-well culture plates and subjected to the different treatments. Following treatment, the media were removed and 20 μl MTT was added to each well. The cells were further cultured at 37°C for 4 h, prior to the removal of the cell culture and the addition of 100 μl dimethyl sulfoxide to dissolve the formazan completely. The absorbance of formazan was determined at 546 nm by the microplate reader and the survival rate was evaluated. All experiments were repeated in triplicate.

### Flow cytometry

HeLa cells were washed three times with phosphate-buffered saline (PBS) and stained with Annexin-V-fluorescein and Puffer (Bio-Rad, Philadelphia, PA, USA), according to the manufacturer’s instructions. Cell fluorescence was determined with a flow cytometer. All experiments were repeated in triplicate.

### Western blot analysis

HeLa cells were collected and lysed with radioimmunoprecipitation assay buffer (Sigma, St. Louis, MO, USA), and the total proteins were evaluated using bicinchoninic acid protein assay reagent (Pierce, Rockford, IL, USA). The protein samples were separated by 10% SDS-PAGE and then transferred to polyvinylidene difluoride membranes. Subsequent to blocking in non-fat dry milk at 37°C for 1 h, the membranes were incubated overnight at 4°C with antibodies against Bcl-2 (1:50), Bax (1:50), Bcl-2 (1:200), cyclin D1 (1:200) and GAPDH (1:500), respectively. The membranes were then washed with PBS with Tween 20 at 10-min intervals, prior to incubation with the secondary diluted anti-mouse or anti-rabbit antibody (1:1,000; Santa Cruz Biotechnology, Inc., Paso Robles, CA, USA) for 1 h at room temperature in the dark. The membranes were developed using enhanced chemiluminescence (Pierce) and exposed to X-ray film. Tanon Gel Imaging System Version 4.00 software (Tanon Science & Technology Co., Ltd., Shanghai, China) was used to analyze the images. The GAPDH was regarded as an internal reference for relative quantification.

### Statistical analysis

All analyses were performed using SPSS statistical software (version 13.0; SPSS Inc., Chicago, IL). Results are expressed as mean ± standard deviation. Significance of difference between the groups was assessed by t-test or ANOVA, and regression analysis was performed. One-tailed P-value of <0.05 was considered as statistically significant.

## Results

### Hsp90 expression in VEGF-C-treated HeLa cells

**Figure 1:**
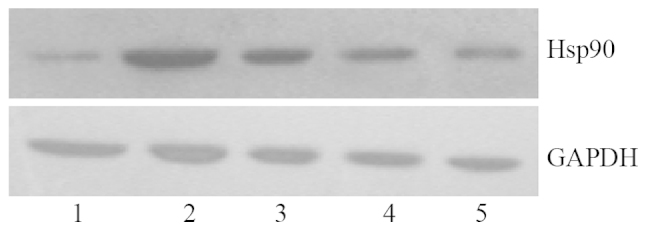
Western blot analysis results of Hsp90 protein expression bands induced by VEGF-C at different times in HeLa cells. Lane 1: Control; Lane 2: VEGF-C (50 ng/μl) 3 h; Lane 3: VEGF-C (50 ng/μl) 6 h; Lane 4: VEGF-C (50 ng/μl) 12 h; Lane 5: VEGF-C (50 ng/μl) 24 h. Hsp90, heat shock protein 90; VEGF-C, vascular endothelial growth factor-C.

VEGF-C was found to induce Hsp90 protein expression in HeLa cells at various treatment time-points, as shown in Table I. Hsp90 protein expression was increased 3.84-fold 3 h following VEGF-C stimulation (1.93±0.17; P<0.05), peaked at 12 h (2.46±0.04; P<0.05) and decreased slightly at 24 h (1.47±0.13; P<0.05). Fig. 1 shows the Hsp90 protein bands in HeLa cells treated with VEGF-C for 3, 6, 12 and 24 h, respectively, using GAPDH as the internal reference.

### Signaling pathways for the induction of Hsp90

**Figure 2:**
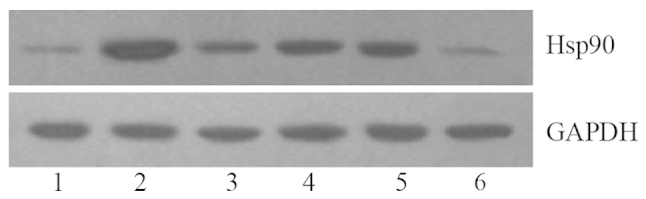
Western blot analysis results of Hsp90 protein expression bands induced by VEGF-C in the presence or absence of various inhibitors in HeLa cells. Lane 1: Control; Lane 2: VEGF-C + KDR-Ab; Lane 3: VEGF-C + PD98059; Lane 4: VEGF-C + LY294002; Lane 5: VEGF-C + GA. Hsp90, heat shock protein 90; GA, geldanamycin; VEGF-C, vascular endothelial growth factor-C.

To investigate whether VEGF-C induced Hsp90 expression via the VEGFR-2 (KDR), MAPK and PI3K pathways, HeLa cells were treated with VEGF-C (50 ng/μl), VEGF-C (50 ng/μl) + KDR-Ab (20 μg/ml), VEGF-C (50 ng/μl) + LY294002 (3 μmol/l), VEGF-C (50 ng/μl) + PD98059 (30 μmol/l) or VEGF-C (50 ng/μl) + GA (10 μmol/l). Results are shown in Table II. Hsp90 expression was increased 3.31-fold in VEGF-C treated HeLa cells; whilst Hsp90 expression was attenuated in the other groups (2.17-, 1.69-, and 1.82-fold in the VEGF-C + KDR-Ab, VEGF-C + PD98059 and VEGF-C + LY294002 groups, respectively; P<0.05), indicating that KDR-Ab, PD98059 and LY294002 partially inhibited Hsp90 expression in HeLa cells induced by VEGF-C. However, there was no significant difference between the expression of Hsp90 between the GA-treated and control cells (0.70±0.05 vs. 0.62±0.21, respectively; P>0.05). Fig. 2 shows the Hsp90 protein bands in HeLa cells following the various treatments, using GAPDH as the internal reference.

### Role of Hsp90 in the proliferation of HeLa cells induced by VEGF-C

GA was demonstrated to completely inhibit the proliferation of HeLa cells induced by VEGF-C. The results from the MTT analysis demonstrated that the proliferation of the HeLa cells was increased ~2.13-fold following treatment with VEGF-C, whilst the proliferation of VEGF-C + GA-treated HeLa cells decreased 0.87-fold (P<0.05; Table III). In addition, the proliferation of HeLa cells treated with GA alone was significantly lower compared with that of control cells (P<0.05), indicating that GA completely inhibited the proliferation of HeLa cells induced by VEGF-C.

### Effect of Hsp90 on the apoptosis inhibition of HeLa cells induced by VEGF-C

The early apoptosis index was determined using annexin V-FITC/PI double staining, and the results are shown in Table III. There was a significant difference in early apoptosis index between the HeLa cells treated with VEGF-C and those treated with VEGF-C + GA (3.29±0.35 versus 6.06±0.78, respectively; P<0.05), and the early apoptosis index of HeLa cells treated with GA alone was significantly higher compared with that of the control cells (10.46±1.12 versus 7.44±0.54, respectively; P<0.05), indicating that GA induced apoptosis of HeLa cells. The results indicate that Hsp90 works in association with VEGF-C in regulating the apoptosis of HeLa cells.

### Role of Hsp90 in the Bcl-2/Bax protein expression induced by VEGF-C

**Figure 3:**
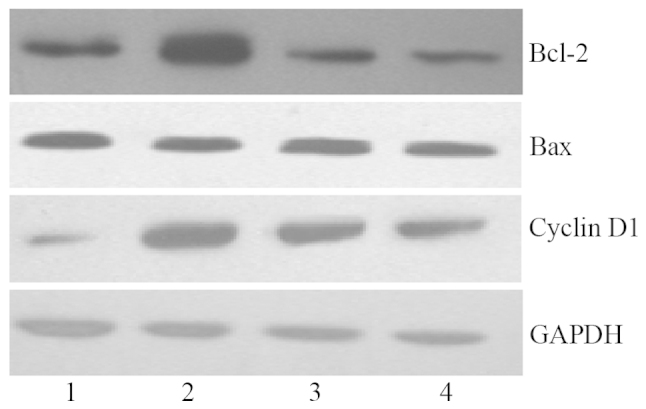
Western blot analysis results of Bcl-2, Bax and cyclin D protein expression bands in HeLa cells. Lane 1: Control; Lane 2: VEGF-C; Lane 3: VEGF-C + GA; Lane 4: GA. Bcl-2, B-cell lymphoma 2; Bax, Bcl-2-associated X protein; GA, geldanamycin; VEGF-C, vascular endothelial growth factor-C.

HeLa cells were incubated without serum for 24 h, and then treated with VEGF-C (50 ng/μl) and VEGF-C (50 ng/μl) + GA (0.02 μmol/l) for 24 h. Bcl-2 and Bax protein expression levels were then determined by western blot analysis. Bcl-2 protein expression was found to be significantly lower in VEGF-C + GA treated HeLa cells than in VEGF-C treated HeLa cells (0.87±0.23 versus 1.90±0.15, respectively; P<0.05), and Bcl-2 protein expression following treatment with GA was significantly lower compared with that in the control cells (0.67±0.16 versus 0.97±0.07, respectively; P<0.05), indicating that even a low concentration of GA (0.02 μmol/l) is able to inhibit the Bcl-2 protein expression induced by VEGF-C. However, there were no significant differences in the levels of Bax protein expression induced by GA, VEGF-C and VEGF-C + GA (P>0.05). In addition, the Bcl-2/Bax expression ratio was lowest in the GA group, followed by the VEGF-C + GA and VEGF-C groups (P<0.05), indicating that even a low concentration of GA had a marked inhibitory effect on Bcl-2 expression. All these results suggest that Hsp90 works in association with VEGF-C in regulating the apoptosis of HeLa cells. The Bcl-2 and Bax protein bands in HeLa cells are shown in Fig. 3, with GAPDH as the internal reference.

### Role of Hsp90 in cyclin D1 protein expression induced by VEGF-C

GA was found to significantly inhibit the cyclin D1 protein expression induced by VEGF-C (Table IV). Cyclin D1 protein expression was significantly lower in VEGF-C + GA-treated HeLa cells than in VEGF-C-treated HeLa cells (0.64±0.21 versus 1.30±0.13, respectively; P<0.05), and cyclin D1 protein expression was significantly lower in GA-treated HeLa cells than in control cells (0.42±0.11 versus 0.57±0.07; P<0.05). These results indicate that even a low concentration of GA had a marked inhibitory effect on cyclin D1 expression, and this suggests that Hsp90 has a role in the induction of cyclin D1 expression by VEGF-C. Cyclin D1 protein bands in HeLa cells are shown in Fig. 3, with GAPDH as the internal reference.

## Discussion

Molecular chaperone Hsp90 is not only of major current interest in fundamental biological research, but is also a target for the treatment of cancer and other diseases. Hsp90 guides the normal folding, intracellular localization and proteolytic turnover of a number of key regulators for cell growth, differentiation and survival (9). Inhibition of Hsp90 function has been shown to cause degradation of target proteins via the ubiquitin proteasome pathway, resulting in the simultaneous depletion of multiple oncoproteins, combinatorial downregulation of signals being propagated via numerous signaling pathways, and modulation of all aspects of the malignant phenotype (10,11). The PI3K and MAPK pathways are involved in the proliferation and apoptosis of HeLa cells induced by VEGF-C, with the overexpression of several downstream genes, including Bcl-2 and cyclin D1. The aim of the present study was to investigate the effect of Hsp90-specific inhibitor GA and VEGF-C on the expression of Hsp90 in HeLa cells.

The effect of Hsp90 and Hsp90-specific inhibitor GA on the proliferation and apoptosis of HeLa cells was investigated. Hsp90 binds to a number of signaling proteins, including ligand dependent transcription factors (e.g., steroid receptor), ligand-independent transcription factors (e.g., MyoD), tyrosine kinases (e.g., v-Src) and serine/threonine kinases (e.g., Raf-1). The role of Hsp90 is to promote the conformational maturation of these receptors and signal-transducing kinases. It interacts with proteins that have already attained a high degree of tertiary structure, and appears to be involved in the maturation and activation of these target proteins rather than their initial folding. Hsp90 chaperone activity depends on its ability to bind and hydrolyze ATP (12,13), which drives a molecular clamp via transient dimerization of the N-terminal domains. HSP90 expression has been shown to be increased in cancer cells (14). It interacts with the signaling proteins to maintain the normal structure and functions of these proteins, and has an important role in the development of tumors (15).

The association between Hsp90 and the proliferation and apoptosis of tumor cells has been investigated in numerous studies. Hsp90 may be involved in the proliferation and apoptosis of tumor cells via the PI3K-AKT/PKB and RAS-RAF-MEK-ERK1/2 pathways (16). Inhibition of Hsp90 function may downregulate Akt kinase, dephosphorylate extracellular signal-regulated kinase and induce cell cycle arrest and cell death (17,18). At present, a number of Hsp90 molecular chaperones have been identified with possible implications on the proliferation and apoptosis of tumor cells, including Bcl-2, AKT/PKB, survivin, c-Raf, JNK, pp60 (v-src), Bcr-Abl, mutant p53, ErbB2 (Her-2), Flt3, HIF-1α, B-Raf and CDK4 (19,20).

GA is a naturally occurring benzoquinone ansamycin, which binds specifically to the N-terminal ATP binding domain of Hsp90 (21), and causes the destabilization and degradation of numerous Hsp90 target proteins. GA specifically inhibits Hsp90 by binding to the ATP hydrolysis site with an affinity >500-times greater than for ATP, thus effectively displacing ATP and disrupting Hsp90-substrate interactions. This makes GA an important candidate in the study of Hsp90 function (22). In a previous study, Duus *et al* (23) investigated Hsp90 expression in a myeloma cell line (U266) using immunofluorescence and flow cytometric analysis, and the results demonstrated that GA treatment resulted in a significant increase in apoptosis and reduction in Bcl-2 expression levels. The Bcl-2-binding protein BAG-1 binds to Bcl-2, Raf-1 kinase and growth factor receptors to inhibit the apoptosis of cells. BAG-1 also binds to steroid hormone receptors associated with Hsp family members.

In the present study, whether Hsp90 is involved in the proliferation and apoptosis of HeLa cells was investigated. *In vitro* treatment of HeLa cells with GA leads to the inhibition of cell proliferation, an exponential increase in apoptosis and a reduction in Bcl-2 expression, indicating that Hsp90 has an important role in the proliferation and apoptosis of cervical carcinoma cells by regulating Bcl-2 expression. However, treatment with GA does not affect Hsp90 expression, indicating that GA downregulates Bcl-2 expression, not by inhibiting Hsp90 mRNA or protein expression, but by inhibiting Hsp90 function. GA may inhibit the binding of Hsp90 to Bcl-2, promoting apoptosis and mediating the signaling pathways for the apoptosis of cervical carcinoma cells. Consequently, it has an important role in the proliferation and apoptosis escape of cervical carcinoma cells.

The association between VEGF-C and Hsp90 was also investigated in the present study. Whether VEGF-C induces Hsp90 expression was investigated. The results of the western blot analysis revealed that Hsp90 protein expression in HeLa cells was induced by VEGF-C when treated for different periods of time. Hsp90 protein expression was increased 3.84-fold following 3 h of VEGF-C stimulation, peaked at 12 h and decreased slightly after 24 h, indicating that VEGF-C induced Hsp90 expression.

In order to investigate whether VEGF-C induced Hsp90 expression via VEGFR-2 (KDR), MAPK and PI3K pathways, HeLa cells were treated with VEGF-C, VEGF-C + KDR-Ab, VEGF-C + LY294002, VEGF-C + PD98059 and VEGF-C + GA. It was found that Hsp90 expression was increased 3.31-fold in VEGF-C treated HeLa cells, and was attenuated in other treatment groups (2.17-, 1.69-, 1.82-fold in VEGF-C + KDR-Ab, VEGF-C + PD98059 and VEGF-C + LY294002, respectively). However, there was no significant difference between the GA-treated cells and control cells (P>0.05). These results indicate that GA functions not by inhibiting Hsp90 mRNA or protein expression, but by inhibiting Hsp90 function. VEGF-C may induce Hsp90 expression via the PI3K and MAPK pathways. In VEGFR-2 (KDR) positive HeLa cells, VEGF-C actives PI3K/AKT and ERK/MAPK pathways via the KDR receptors, and upregulates Hsp90 expression.

The role of Hsp90 in the proliferation and apoptosis of HeLa cells induced by VEGF-C was also investigated in the present study. The Hsp90-specific inhibitor GA was found to completely inhibit the proliferation of HeLa cells induced by VEGF-C. The proliferation of the VEGF-C treated HeLa cells was increased ~2.13-fold, whereas that of the VEGF-C + GA treated HeLa cells decreased 0.87-fold (P<0.05). The proliferation of GA-treated HeLa cells was significantly lower compared with that of control cells (P<0.05). These results indicate that Hsp90 participates in the VEGF-C induced proliferation and apoptosis of HeLa cells. In addition, it was shown that even a low concentration of GA (0.02 μmol/l) inhibits the Bcl-2 and cyclin D1 protein expression induced by VEGF-C, but appears to have no effect on Bax protein expression.

In the present study, VEGF-C was indicated to induce Hsp90 expression via the PI3K and MAPK pathways. Hsp90 binds to a number of specific signaling proteins that require this interaction to execute their function, and upregulates the expression of downstream genes, including Bcl-2 and cyclin D1. Therefore, Hsp90 has a critical role in the proliferation and apoptosis of HeLa cells. Hsp90 modulates Bcl-2 expression, as shown by the complete inhibition of VEGF-induced Bcl-2 expression and binding to Hsp90 by Hsp90-specific inhibitor GA; VEGF has been shown to promote the survival of leukemic cells by the Hsp90-mediated induction of Bcl-2 expression and apoptosis inhibition (23). Typical Hsp90 target proteins include Bcl-2, AKT/PKB, c-Raf, B-Raf and CDK4 (19). Whether other target proteins are involved requires further investigation.

## Figures and Tables

**Table I:** Heat shock protein 90 expression in VEGF-C treated HeLa cells.

Treatment duration	Expression^a^	Ratio^b^	P-value
0 h	0.50±0.07	1	
3 h	1.93±0.17	3.84	<0.05^c^
6 h	2.08±0.26	4.15	<0.05^c^,^d^
12 h	2.46±0.04	4.9	<0.05^c^,^d^
24 h	1.47±0.13	2.92	<0.05^c^,^d^

a Mean ± SD;

b relative to the control (0 h);

c P<0.05, compared with the control;

d P<0.05, compared with the previous time-point.

VEGF-C, vascular endothelial growth factor-C.

**Table II:** Effect of inhibitors of various signaling pathways on the induction of heat shock protein 90 by VEGF-C.

Treatment	Expression^a^	Ratio^b^	P-value
Control	0.62±0.21	1	
VEGF-C	2.04±0.15	3.31	<0.05^c^
VEGF-C+KDR-Ab	1.33±0.26	2.17	<0.05^d^
VEGF-C+LY294002	1.04±0.08	1.69	<0.05^d^
VEGF-C+PD98059	1.11±0.11	1.82	<0.05^d^
VEGF-C+GA	0.70±0.05	1.14	>0.05^c^

a Mean ± SD;

b relative to the control;

c P<0.05, compared with the control;

d P<0.05, compared with VEGF-C treatment.

VEGF-C, vascular endothelial growth factor-C; GA, geldanamycin.

**Table III:** Effect of VEGF-C and GA on the proliferation and apoptosis of HeLa cells.

	Proliferation			Early apoptosis index		
		
Treatment	Absorbance^a^	Proliferation index	P-value	Apoptosis index^a^	Relative ratio^b^	P-value
Control	0.62±0.02	1		7.44±0.54	1	
VEGF-C	1.32±0.09	2.13	<0.05^c^	3.29±0.35	0.44	<0.05^c^
VEGF-C+GA	0.54±0.02	0.87	<0.05^c^	6.06±0.78	0.81	<0.05^d^
GA	0.46±0.18	0.74	<0.05^c^,^d^	10.46±1.12	1.41	<0.05^c^

a Mean ± SD;

b relative to the control;

c P<0.05, compared with the control;

d P<0.05, compared with VEGF-C treatment.

VEGF-C, vascular endothelial growth factor-C; GA, geldanamycin.

**Table IV:** Effect of GA on Bcl-2, Bax and cyclin D1 protein expression induced by VEGF-C.

	Bcl-2			Bax			Bcl-2/Bax			Cyclin D1		
				
Treatment	Expression^a^	Ratio	P-value	Expression^a^	Ratio	P-value	Expression^a^	Ratio	P-value	Expression^a^	Ratio	P-value
Control	0.97±0.07	1		0.70±0.09	1		1.38±0.09	1		0.57±0.07	1	
V	1.90±0.15	2.73	<0.05^b^,^c^	0.79±0.14	1.13	>0.05^b^	2.40±0.15	1.74	<0.05^b^	1.30±0.13	2.29	<0.05^b^
V+GA	0.87±0.23	1.28	<0.05^c^	0.68±0.13	0.97	>0.05^b^	1.28±0.20	0.93	<0.05^c^	0.64±0.21	1.13	<0.05^c^
GA	0.67±0.16	0.74	<0.05^b^,^c^	0.90±0.16	1.30	>0.05^b^	0.74±0.16	0.54	<0.05^b^,^c^	0.42±0.11	0.76	<0.05^b^,^c^

a Mean ± SD;

b P<0.05, compared with the control;

c P<0.05, compared with VEGF-C treatment.

V, vascular endothelial growth factor-C (VEGF-C); GA, geldanamycin; Bcl-2, B-cell lymphoma 2; Bax, Bcl-2-associated X protein.

## References

[1] Bishop SC, Burlison JA, Blagg BS (2007). Hsp90: a novel target for the disruption of multiple signaling cascades. Curr Cancer Drug Targets.

[2] Trepel J, Mollapour M, Giaccone G, Neckers L (2010). Targeting the dynamic HSP90 complex in cancer. Nat Rev Cancer.

[3] Chiosis G, Dickey CA, Johnson JL (2013). A global view of Hsp90 functions. Nat Struct Mol Biol.

[4] Lee WY, Chen YC, Shih CM (2014). The induction of heme oxygenase-1 suppresses heat shock protein 90 and the proliferation of human breast cancer cells through its byproduct carbon monoxide. Toxicol Appl Pharmacol.

[5] Schwock J, Pham NA, Cao MP, Hedley DW (2008). Efficacy of Hsp90 inhibition for induction of apoptosis and inhibition of growth in cervical carcinoma cells in vitro and in vivo. Cancer Chemother Pharmacol.

[6] Neckers L (2006). Chaperoning oncogenes: Hsp90 as a target of geldanamycin. Handb Exp Pharmacol.

[7] Mehta PP, Kung PP, Yamazaki S (2011). A novel class of specific Hsp90 small molecule inhibitors demonstrate in vitro and in vivo anti-tumor activity in human melanoma cells. Cancer Lett.

[8] Du X, Mi R (2011). Study on the cell signal transductions of cell proliferation and apoptosis induced by VEGF-C in cervical carcinoma. Xian Dai Fu Chan Ke Za Zhi.

[9] Chinnaiyan P, Allen GW, Harari PM (2006). Radiation and new molecular agents, part II: targeting HDAC, HSP90, IGF-1R, PI3K, and Ras. Semin Radiat Oncol.

[10] Park JW, Yeh MW, Wong MG (2003). The heat shock protein 90-binding geldanamycin inhibits cancer cell proliferation, down-regulates oncoproteins, and inhibits epidermal growth factor-induced invasion in thyroid cancer cell lines. J Clin Endocrinol Metab.

[11] Sreedhar AS, Kalmár E, Csermely P, Shen YF (2004). Hsp90 isoforms: functions, expression and clinical importance. FEBS Lett.

[12] Schmitt E, Gehrmann M, Brunet M, Multhoff G, Garrido C (2007). Intracellular and extracellular functions of heat shock proteins: repercussions in cancer therapy. J Leukoc Biol.

[13] Polier S, Samant RS, Clarke PA (2013). ATP-competitive inhibitors block protein kinase recruitment to the Hsp90-Cdc37 system. Nat Chem Biol.

[14] Richter K, Buchner J (2001). Hsp90: chaperoning signal transduction. J Cell Physiol.

[15] Cappello F, David S, Rappa F (2005). The expression of HSP60 and HSP10 in large bowel carcinomas with lymph node metastase. BMC Cancer.

[16] Powers MV, Workman P (2006). Targeting of multiple signalling pathways by heat shock protein 90 molecular chaperone inhibitors. Endocr Relat Cancer.

[17] Georgakis GV, Li Y, Rassidakis GZ (2006). Inhibition of heat shock protein 90 function by 17-allylamino-17-demethoxy-geldanamycin in Hodgkin’s lymphoma cells down-regulates Akt kinase, dephosphorylates extracellular signal-regulated kinase, and induces cell cycle arrest and cell death. Clin Cancer Res.

[18] Mitsiades CS, Mitsiades NS, McMullan CJ (2006). Antimyeloma activity of heat shock protein-90 inhibition. Blood.

[19] Ferrario A, Rucker N, Wong S, Luna M, Gomer CJ (2007). Survivin, a member of the inhibitor of apoptosis family, is induced by photodynamic therapy and is a target for improving treatment response. Cancer Res.

[20] Röhl A, Rohrberg J, Buchner J (2013). The chaperone Hsp90: changing partners for demanding clients. Trends Biochem Sci.

[21] Sidera K, Patsavoudi E (2014). HSP90 Inhibitors: current development and potential in cancer therapy. Recent Pat Anticancer Drug Discov.

[22] Gooljarsingh LT, Fernandes C, Yan K (2006). A biochemical rationale for the anticancer effects of Hsp90 inhibitors: slow, tight binding inhibition by geldanamycin and its analogues. Proc Natl Acad Sci USA.

[23] Duus J, Bahar HI, Venkataraman G (2006). Analysis of expression of heat shock protein-90 (HSP90) and the effects of HSP90 inhibitor (17-AAG) in multiple myeloma. Leuk Lymphoma.

